# Constitutive Activation of STAT3 in Myeloma Cells Cultured in a Three-Dimensional, Reconstructed Bone Marrow Model

**DOI:** 10.3390/cancers10060206

**Published:** 2018-06-16

**Authors:** Yung-Hsing Huang, Ommoleila Molavi, Abdulraheem Alshareef, Moinul Haque, Qian Wang, Michael P. Chu, Christopher P. Venner, Irwindeep Sandhu, Anthea C. Peters, Afsaneh Lavasanifar, Raymond Lai

**Affiliations:** 1 Department of Laboratory Medicine and Pathology, University of Alberta, Edmonton, AB T6G 2R3, Canada; yunghsin@ualberta.ca (Y-H.H.); omolavi@ualberta.ca (O.M.); al15@ualberta.ca (A.A.); moinul@ualberta.ca (M.H.); qw1@ualberta.ca (Q.W.); 2 Faculty of Pharmacy, Tabriz University of Medical Science, Tabriz P.O.Box 51664-14766, East Azerbaijan Province, Iran; 3 Department of Oncology, University of Alberta, Edmonton, AB T6G2R7, Canada; mpchu@ualberta.ca (M.P.C.); cvenner@ualberta.ca (C.P.V.); irwindee@ualberta.ca (I.S.); anthea1@ualberta.ca (A.C.P.); 4 Department of Medicine, University of Alberta, Edmonton, AB T6G2G3, Canada; 5 Faculty of Pharmacy and Pharmaceutical Sciences, University of Alberta, Edmonton, AB T6G2H7, Canada; afsaneh@ualberta.ca

**Keywords:** 3D culture, multiple myeloma, STAT, bortezomib, CETSA, Stattic

## Abstract

Malignant cells cultured in three-dimensional (3D) models have been found to be phenotypically and biochemically different from their counterparts cultured conventionally. Since most of these studies employed solid tumor types, how 3D culture affects multiple myeloma (MM) cells is not well understood. Here, we compared MM cells (U266 and RPMI8226) in a 3D culture model with those in conventional culture. While the conventionally cultured cells were present in single cells or small clusters, MM-3D cells grew in large spheroids. We discovered that STAT3 was the pathway that was more activated in 3D in both cell lines. The active form of STAT3 (phospho-STAT3 or pSTAT3), which was absent in MM cells cultured conventionally, became detectable after 1–2 days in 3D culture. This elevated pSTAT3 level was dependent on the 3D environment, since it disappeared after transferring to conventional culture. STAT3 inhibition using a pharmacological agent, Stattic, significantly decreased the cell viability of MM cells and sensitized them to bortezomib in 3D culture. Using an oligonucleotide array, we found that 3D culture significantly increased the expression of several known STAT3 downstream genes implicated in oncogenesis. Since most primary MM tumors are naturally STAT3-active, studies of MM in 3D culture can generate results that are more representative of the disease.

## 1. Introduction

Studies of malignant cells using three-dimensional (3D) culture systems are believed to provide information that is more representative of the ‘real-life’ in vivo conditions, as opposed to those using cells cultured conventionally in monolayer or cell suspension. In keeping with this concept, malignant cells cultured in 3D have been shown to display substantial differences in their growth characteristics, gene expression and drug resistance patterns when compared to cells cultured conventionally [1,2,3]. Importantly, cells grown in biomimetic 3D systems are phenotypically similar to tumors formed in vivo. In one study, unlike their counterparts cultured in monolayer, glioblastoma cells cultured in 3D were found to phenotypically mimic xenografts formed in mice, with respect to their growth rate, levels of hypoxia and angiogenesis [4]. Similarly, in another study, it was found that the drug resistance profile of glioblastoma cell lines derived from patient-derived xenografts correlates with the clinical outcome of these patients, and the correlations were better than that of cells cultured conventionally [5]. From our literature search, we have identified a good number of studies employing various 3D models to study cancer biology, with the majority of these studies focusing on malignant epithelial cells and neurogenic cells. In comparison, studies of malignant hematopoietic cells using 3D culture models are relatively scarce, and the impact of 3D culture on these cancer cells is incompletely understood. 

Multiple myeloma (MM), characterized by the accumulation of clonal malignant plasma cells in the three-dimensional bone marrow niches, represents 10% of all hematologic malignancies [6]. Although the recent advances in various therapeutic modalities have improved the 5-year survival of MM patients to ~50%, MM remains to be an incurable disease [7,8]. The tumor microenvironment within the bone marrow niche is believed to play an essential role in the development and progression of MM. For example, it was found that vascular endothelial growth factor secreted by MM cells can induce the release of IL-6 from bone marrow stromal cells, which in turn promotes the proliferation and survival of MM cells [9]. In light of the importance of the microenvironment, several animal models have been developed to study the biology of MM and to evaluate various therapeutics designed to treat MM [10,11,12]. Nonetheless, to our knowledge, studies of MM using 3D models are relatively few [13]. For example, Ferrarini et al. employed a bioreactor system to create the 3D condition, although this bioreactor is relatively expensive and thus, not widely accessible [14]. De la Puente et al. employed cross-linked fibrinogen matrix supplemented with patient-derived mononuclear cells and supernatants [15]. Kirshner et al. described a 3D model in which Matrigel^®^, which is commercially available, was found to support the expansion of primary MM cells for up to 30 days [16]. This 3D model carries several important advantages over animal models, as it is relatively inexpensive and devoid of issues related to cross-species immune incompatibilities. We also believe that Kirshner’s 3D model is more accessible to researchers, as it does not require the purchase of relatively expensive equipment or elaborative preparation of patient samples. Nonetheless, how exactly the 3D culture impacts the biology of MM cells is largely unknown. 

To evaluate the impact of the 3D culture on MM cells, we optimized a 3D reconstructed bone marrow model based on the method previously described by Kirshner et al. [16,17]. The modifications to the system have generated several improvements, such as the fact that our system is highly amendable to histologic processing, immunocytochemical studies and possibly other morphologic studies (i.e., studies of cell-cell interactions). Importantly, our results have highlighted the importance of STAT3, which was found to be active in MM-3D cells but not those cultured conventionally. Our data supports the concept that STAT3 increases the expression of proteins which are responsible for enhanced cell survival, proliferation and drug resistance in MM [18,19,20,21]. As STAT3 is often active in primary MM cells [22], we believe that studies of MM in the 3D culture systems can generate results that are more representative of the disease.

## 2. Results

### 2.1. MM Cells Cultured in 3D Form Large Clusters

**Figure 1:**
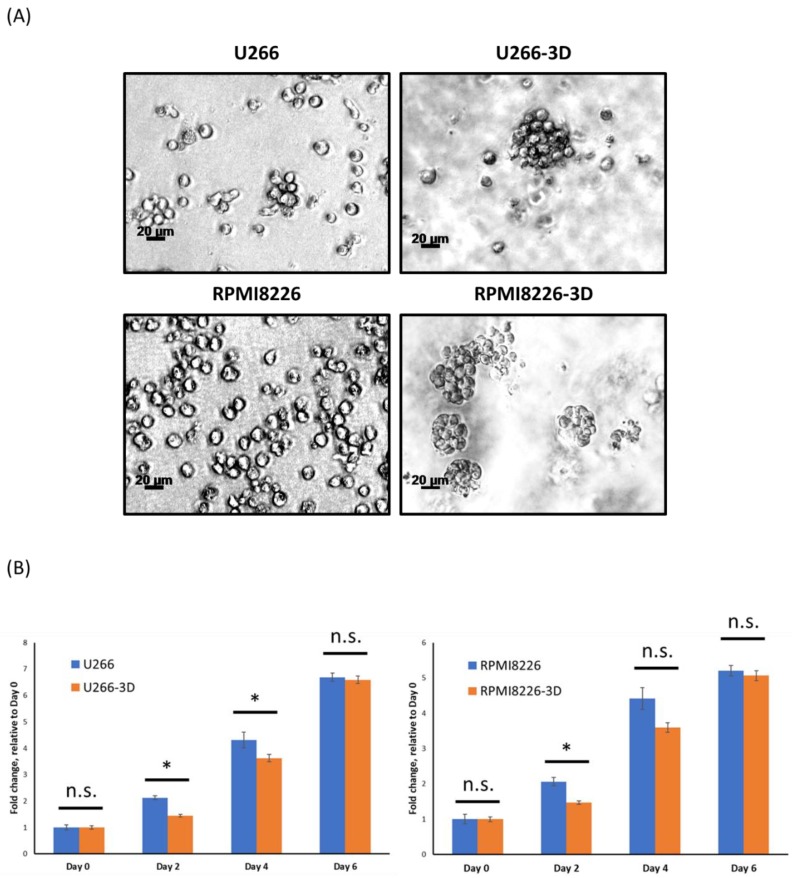
MM cells exhibit different appearances and growth patterns in conventional culture versus in 3D culture. (**A**) The morphology of U266 and RPMI8226 cells in conventional or 3D culture after 6 days was examined by phase contrast microscopy. Images were taken at 100X magnification. A scale bar equivalent to 20 µm is included in each graph; (**B**) The growth of U266 and RPMI8226 cells in conventional (blue bars) or 3D cultures (orange bars) was measured by the trypan blue exclusion assay at various time points. Fold changes of total viable cells were normalized to the cell number on day 0 (2.5 × 10^5^ cells). The error bars represent standard deviation from a triplicate experiment, * *p* < 0.05, n.s. not significant, Student’s *t*-test.

We cultured two MM cell lines, U266 and RPMI8226. using the 3D model that had been optimized, as described in Materials and Methods [17]. These cells were labeled MM-3D cells, and we compared their growth characteristics with cells cultured conventionally. As shown in Figure 1A, MM cells from both cell lines cultured conventionally settled in the bottom of the tissue culture flasks, and they were found to be present in small clusters composed of an average of 5–10 cells with a greatest dimension of 20–30 µm (i.e., U266) or predominantly in single cells (i.e., RPMI8226). In contrast, MM-3D cells from both cell lines were present predominantly as spherical, tight cell clusters that were composed of >20–30 cells with the greatest dimension of >50 µm (*p* < 0.05, Figure S1). We then compared the cell growth in these two different culture conditions using the trypan blue exclusion assay. As shown in Figure 1B, we found that MM-3D cells grew significantly slower than those cultured conventionally in the first few days of culture (*p* < 0.05), although the differences were relatively small. These differences in cell growth became statistically insignificant on day 4 for RPMI8226 and on day 6 for U266.

### 2.2. STAT3 Activity in MM Cells is Increased in 3D Culture

**Figure 2:**
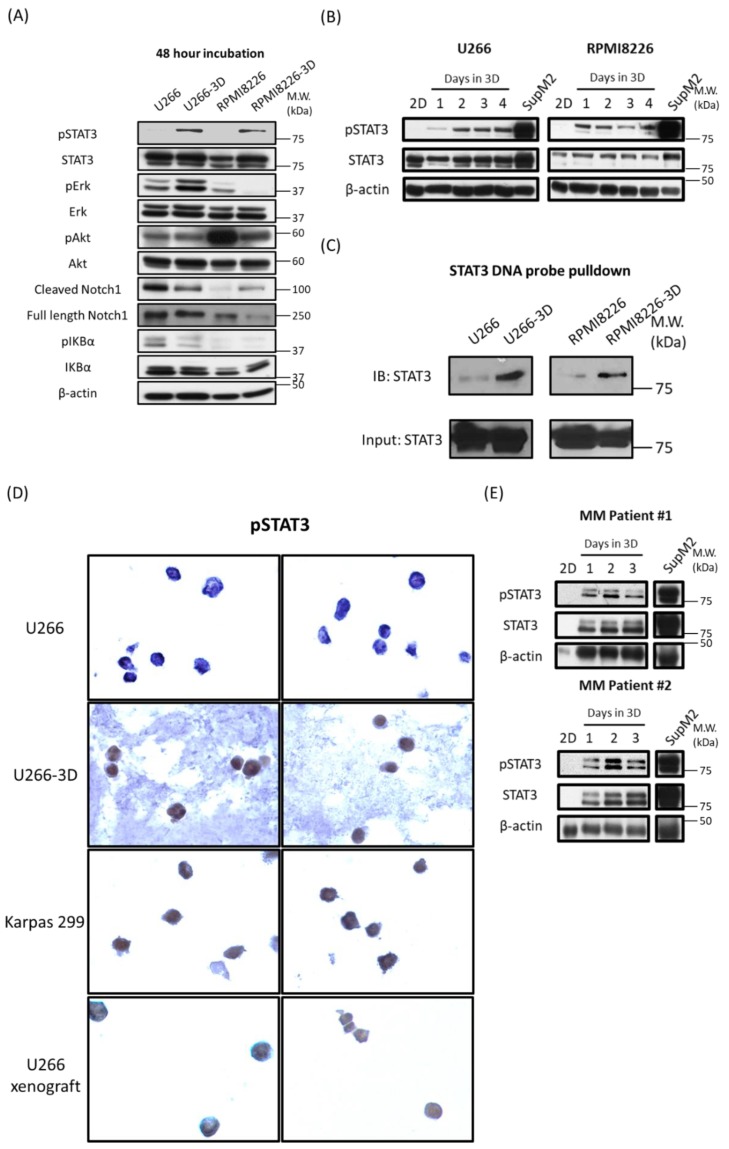
MM cells cultured in 3D acquire STAT3 activity. (**A**) The activity of various signaling pathways (STAT3, Erk/MAPK, PI3K/Akt, NF-κB and Notch) in U266 and RPMI8226 cells cultured conventionally (2D) or in 3D was examined by Western blot analysis after 48 h; (**B**) The STAT3 activity of U266 and RPMI8226 cells in 2D or 3D culture from day 1 to day 4 were examined by Western blot analysis of pSTAT3 levels. SupM2 cells were included as a positive control for the pSTAT3 level; (**C**) The DNA binding ability of STAT3 in U266 and RPMI8226 cells cultured in 2D or 3D was examined by DNA pulldown immunoblotting assay. The cells were harvested and lysed after 48 h in culture. STAT3 in cell lysate was pulled down by a STAT3 DNA probe (described in Materials and Methods); (**D**) Immunocytochemical analysis of pSTAT3 level in U266, U266-3D, Karpas 299 and U266 xenograft cells. The cells were fixed after 48 h in culture. The procedure of processing, embedding and sectioning was described in Materials and Methods. Two representative pictures were shown. Karpas 299 cells were included as a positive control for pSTAT3 staining; (**E**) Western blot analysis of pSTAT3 and STAT3 levels of primary MM bone marrow cells in 2D or 3D culture from day 1 to day 3. SupM2 cells were included as a positive control for pSTAT3 level. β-actin was probed as a loading control in each blot.

Deregulations of several signaling pathways, including that of STAT3, Erk/MAPK, PI3K/Akt, NF-κB and Notch, are known to be important in the pathogenesis of MM [22,23,24,25,26,27,28]. To determine if 3D culture has a significant impact on the cellular signaling in MM cells, we examined the status of these 5 pathways in U266 and RPMI8226 cells, cultured in 3D or conventionally. As shown in Figure 2A, using lysates prepared from cells harvested on day 2, we found that the active/phosphorylated form of STAT3 (pSTAT3) was expressed in MM-3D cells, whereas this band was not detectable in cells cultured conventionally. 

We did not observe consistent and/or obvious difference in the activation status of the other 4 signaling pathways (Figure 2A). In view of these findings, we focused our studies on STAT3. We then performed a time course experiment to study the kinetics of STAT3 activation in MM-3D cells. Cells from both MM cell lines were cultured in 3D for 4 days and the expression level of pSTAT3 was examined daily using Western blot analysis. Triplicate experiments were performed and results from a representative experiment are shown in Figure 2B. In U266 cells, the pSTAT3 band became detectable on day 1, and there was a time-dependent upregulation of pSTAT3 which peaked on day 4. In RPMI8226 cells, the pSTAT3 band also became detectable on day 1 and but it appeared to diminish gradually thereafter. In comparison, no pSTAT3 band was detectable in cells cultured conventionally throughout the experiment (Figure S2). Cell lysates from SupM2, an ALK-positive anaplastic large cell lymphoma cell line known to have a high pSTAT3 expression [29], were used as the positive control. To explore the possible activators of STAT3 in 3D culture, we checked the expression level of several cytokines which are known to induce STAT3 phosphorylation in MM: IL6, IL21 and IL10 [30,31,32]. As shown in Figure S3, the expression of all three cytokines in U266 cells increased by 1.5–2.5 folds after 1 day of 3D culture compared to cells in conventional culture.

In support of the concept that the STAT3 transcriptional activity was indeed increased in MM-3D cells, we examined the DNA-binding ability of STAT3 using the protein-DNA pulldown assay. As shown in Figure 2C, a substantially high level of STAT3 protein in MM-3D cells was pulled down with the biotinylated DNA probe containing the STAT3 consensus sequence; in comparison, no band was detectable when cells cultured conventionally were examined. To estimate the proportion of MM-3D cells showing pSTAT3 expression, we optimized our experimental protocol, as detailed in Materials and Methods, so that MM-3D cells and the surrounding matrix were readily fixed in formalin and processed for immunocytochemistry. As shown in Figure 2D, the vast majority of U266 cells cultured in 3D (over 100 cells examined) showed definitive evidence of nuclear pSTAT3 staining, and this finding suggests that STAT3 activation in 3D culture was a generalized phenomenon and not restricted to a small cell subset. Additionally, a similar STAT3 activation pattern was also observed in the U266 cells xenografted in an animal, suggesting that the 3D culture reflected the in vivo MM condition better than the conventional culture. Lastly, we examined if the cell concentration affects the activation of STAT3 in MM-3D cells. Thus, we doubled the cell density from 2.5 × 10^5^/mL to 5.0 × 10^5^/mL at the beginning of the 3D culture. As shown in Figure S4, while both cell lines acquired pSTAT3 on day 1, this signal decreased with time and became undetectable on day 3 or day 4. This time-dependent decrease in pSTAT3 was likely due to the depletion of nutrients in the tissue culture. 

We also had collected evidence that the observed STAT3 activation in MM-3D is not a cell line-specific phenomenon. As shown in Figure 2E, we studied two primary patient bone marrow aspirate samples using western blot analysis, and we found that MM-3D cells, but not cells in conventional culture, showed a substantial level of pSTAT3 expression that peaked on day 2, similar to that seen in U266 cells cultured in 3D. 

### 2.3. STAT3 Activation in MM-3D Cells Is Dependent on the 3D Environment

**Figure 3:**
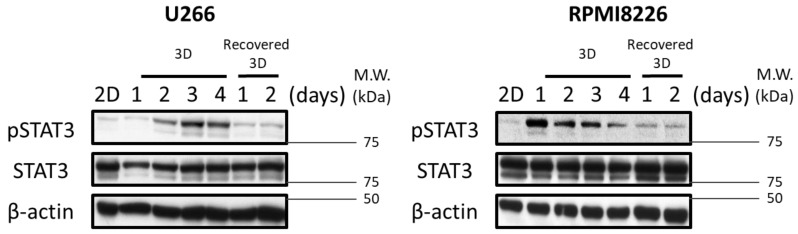
Acquired STAT3 activity in MM cells diminished upon transfer from 3D to conventional culture. The STAT3 activity in U266 and RPMI8226 cells before and after transfer from 3D culture by Western blot analysis of pSTAT3 level. U266 and RPMI8226 were pre-cultured in 3D culture for 2 days and 1 day prior to transfer to reach a substantial pSTAT3 level, respectively. β-actin was probed as a loading control. 2.5 × 10^5^ cells were seeded initially.

To understand if the expression of pSTAT3 is truly dependent on the 3D culture environment, we extracted MM-3D cells from 3D culture matrix and transferred them to conventional cell culture. Specifically, the matrix was dissolved, and pelleted MM-3D cells were washed and re-suspended in growth medium at a cell density of 2.5 × 10^5^ cells/mL. The expression level of pSTAT3 was then evaluated at 24 h and 48 h using Western blot analysis. As shown in Figure 3, the pSTAT3 level substantially decreased on day 2 after transfer to conventional culture in both U266 and RPMI8226 cells.

### 2.4. STAT3 Inhibition Is Effective in Decreasing Cell Growth of MM-3D Cells

**Figure 4:**
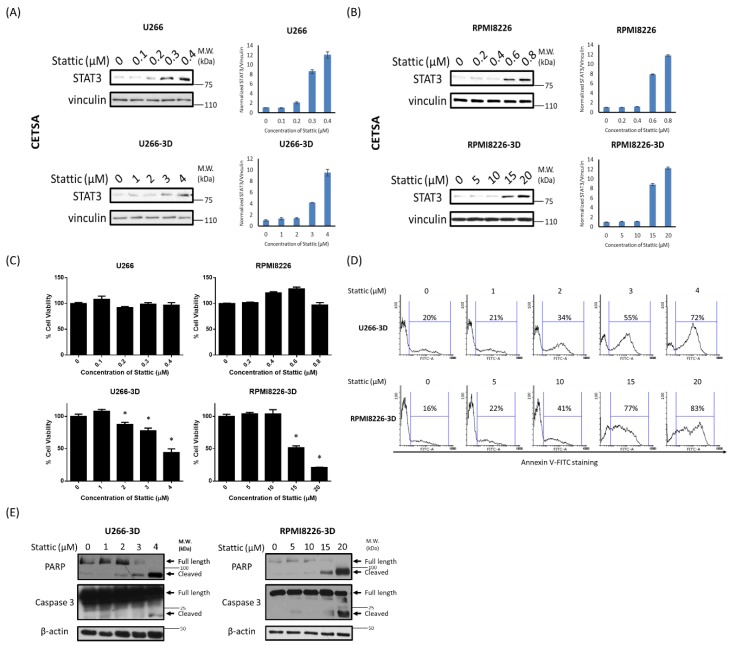
MM cells cultured in 3D are more sensitive to STAT3 inhibition. CETSA of (**A**) U266 and (**B**) RPMI8226 cells in conventional or 3D culture after 1 h of Stattic treatment. Vinculin was blotted as a loading control. The STAT3/vinculin ratios were quantified using ImageJ and shown on the right. Error bars represent the standard deviation from two independent experiments; (**C**) The effect of STAT3 inhibition on cell viability of U266 and RPMI8226 cells in conventional or 3D culture. The cells were treated with Stattic for 24 h. Cell viability was measured by MTS assay and normalized to cells with no Stattic treatment. The error bars represent standard deviation from a triplicate experiment, * *p* < 0.05, Student’s *t*-test; (**D**) The effect of STAT3 inhibition on apoptosis in U266- and RPMI8226-3D cells. The cells were treated with Stattic for 24 h and stained with an apoptotic marker Annexin V. The percentage of Annexin V-positive cells was analyzed by flow cytometry; (**E**) The expression levels of two apoptotic markers, cleaved PARP and cleaved caspase 3, in U266- and RPMI8226-3D cells after 24 h of Stattic treatment were examined by Western blot analysis. β-actin was probed as a loading control. For all the experiments above, U266 and RPMI8226 cells were cultured for 2 and 1 days before the Stattic treatment to reach a substantial pSTAT3 level, respectively. 2.5 × 10^5^ cells were seeded initially.

To investigate the biological significance of 3D-induced STAT3 activation, we inhibited STAT3 using a STAT3 pharmacologic inhibitor, Stattic, which has been extensively described in the literature [33]. Since we anticipated the intracellular drug level will be highly dependent on the types of tissue culture (e.g., cell suspension versus solid matrix), we employed cellular thermal shift assay (CETSA) [34], to compare the extent of STAT3-Stattic binding in MM-3D cells and in cells cultured in suspension. As shown in Figure 4A, in U266-3D cells, Stattic was found to be substantially bound to STAT3 at a dose of 4 µM, which was found to induce more than 50% reduction in cell viability after 24 h (Figure 4C). In comparison, in U266 cells grown conventionally, 0.3 µM of Stattic was the lowest dosage found to be effective in mediating a substantial physical binding between Stattic and STAT3, and this dosage of Stattic did not induce any significant loss of cell viability. Similarly, in RPMI8226-3D cells, a substantial Stattic-STAT3 binding was observed at 15 µM (Figure 4B), which induced more than 50% reduction in cell viability (Figure 4C). In comparison, only 0.6 µM of Stattic was required for a substantial Stattic-STAT3 binding in conventionally cultured cells, and no significant reduction in cell viability was seen at this dosage. In summary, with a comparable level of Stattic-STAT3 binding, MM-3D cells showed significant reduction in cell growth whereas cells in suspension did not show significant changes. These findings support the concept that STAT3 activation in MM-3D is biologically important.

To investigate the mechanism underlying the Stattic-induced reduction in cell viability, we asked if apoptosis played a role. As shown in Figure 4D, Stattic was found to induce apoptosis in MM-3D cells, as shown by the level of Annexin V staining, cleaved PARP and caspase 3 (Figure 4E). Specifically, the expression of cleaved PARP and caspase 3 was detectable at the same dosages of Stattic at which a substantial binding between Stattic-STAT3 was found (i.e., 3–4 µM for U266 cells and 15–20 µM for RPMI8226 cells). In contrast, no sign of apoptosis was observed in both cell lines cultured conventionally; specifically, no appreciable Annexin V staining, cleaved PARP and cleaved caspase 3 were found at the dose range where Stattic can effectively bind to STAT3 (i.e., 0.3–0.4 µM for U266 and 0.6–0.8 µM for RPMI8226) (Figure S5A,B).

### 2.5. STAT3 Inhibition Sensitizes MM-3D Cells to Bortezomib

**Figure 5:**
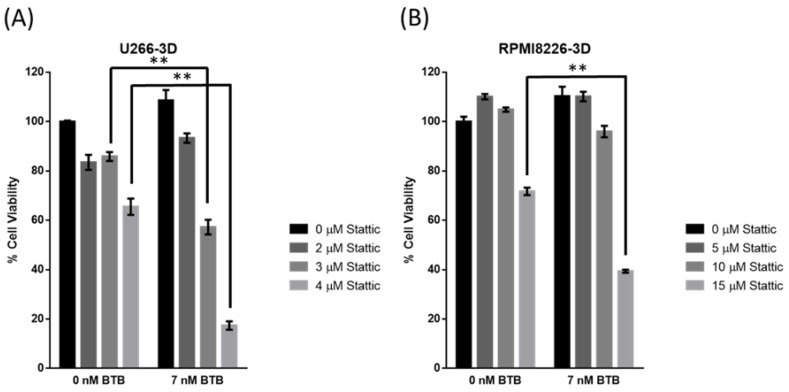
STAT3 inhibition in MM-3D cells sensitizes them to bortezomib. Cell viability of (**A**) U266- and (**B**) RPMI8226-3D cells was measured after treatment with Stattic, bortezomib (BTB) or both for 48 h. U266 and RPMI8226 were pre-cultured in 3D for 2 days and 1 day before drug treatment to reach a substantial pSTAT3 level, respectively. Cell viability was measured by MTS assay and normalized to the cell viability of untreated cells. 2.5 × 10^5^ cells were seeded initially. The error bars represent standard deviation from a triplicate experiment, ** *p* < 0.001, Student’s *t*-test.

Since EGFR-induced STAT3 activation has been shown to promote resistance to proteasome inhibitors in MM cells [21], we asked if the STAT3 activation in MM-3D cells contributes to resistance to bortezomib, a proteasome inhibitor commonly used in treating MM patients. To address this question, we tested if Stattic can sensitize the STAT3-active MM-3D cells to bortezomib-induced cytotoxicity. Thus, we cultured U266 cells in 3D for 48 h, and this resulted in a relatively high expression level of pSTAT3 in these cells (Figure 2). We then treated these cells with bortezomib at dose (i.e., 7 nM) that we had already confirmed to be slightly lower than that of the inhibitory concentration at 50% (IC_50_). For Stattic treatment, we used two doses where substantial Stattic can bind to STAT3, as illustrated in Figure 4A,B. As shown in Figure 5A, treatment with a combination of 7 nM of bortezomib and 3 or 4 µM of Stattic resulted in a significantly higher reduction in the number of viable U266-3D cells, as compared to cells treated with bortezomib or Stattic alone (*p* < 0.001). Similar results were observed for RPMI8226-3D cells (Figure 5B). In contrast, Stattic treatment did not improve the cytotoxic effect of bortezomib to both MM cell lines cultured conventionally (Figure S6).

### 2.6. Gene Expression Profiling in MM-3D Cells

**Figure 6:**
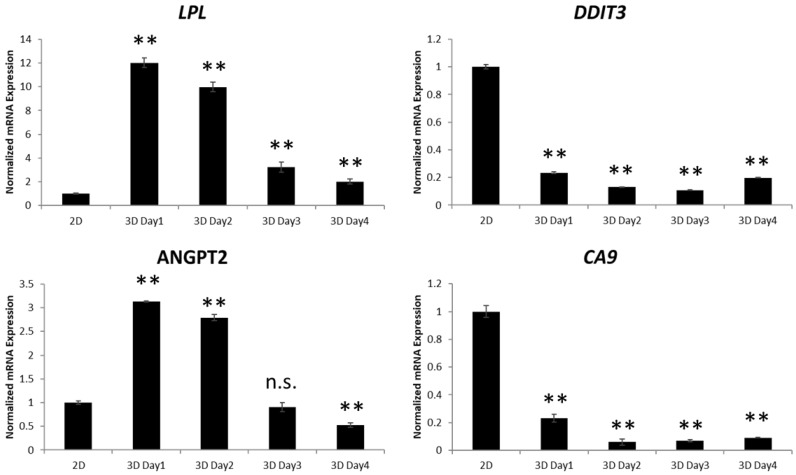
3D culture changes the gene expression in MM cells. Quantitative RT-PCR of *LPL*, *ANGPT2*, *DDIT3* and *CA9* mRNA levels in U266 cells in conventional culture (2D) or day 1 to 4 in 3D culture. 2.5 × 10^5^ cells were seeded initially. The primers used for each gene are shown in Materials and Methods. The error bars represent standard deviation from a triplicate experiment, n.s. not significant and ** *p* < 0.001 compared to 2D, one-way ANOVA with Dunnett’s multiple *t*-test.

To better understand the biochemical changes induced by the 3D culture, we performed an oligonucleotide array comparing U266-3D and U266 cultured conventionally. The RT^2^ Profiler Human Cancer PathwayFinder PCR Array containing 90 genes implicated in oncogenesis was used, as detailed in Materials and Methods. Compared to U266 cells grown conventionally, U266-3D cells showed an increase in the expression of lipoprotein lipase (*LPL*, 14.1 folds), angiopoietin 2 (*ANGPT2*, 6.8 folds) and Snail homolog 3 (*SNAI3*, 3.5 folds), and a decrease in the expression of DNA-damage-inducible transcript 3 (*DDIT3*, -35.9 folds), carbonic anhydrase 9 (*CA9*, −22.3 folds) and ovalbumin (*SERPINB2*, −19.4 folds) (Figure S7A). By performing signaling pathway analysis using Pathway Common Network Visualizer (*www.pathwaycommons.org/pcviz*), we found that 4 out of these 6 most modulated genes (*LPL, ANGPT2, DDIT3 and CA9*) are directly or indirectly related to STAT3 (Figure S7B). The upregulation of *LPL* and *ANGPT2* and downregulation of *DDIT3* and *CA9* in 3D culture were confirmed by quantitative RT-PCR (Figure 6C). Specifically, the mRNA levels of *LPL* and *ANGPT2* increased by approximately 10 and 2.8 folds on day 2 in 3D culture compared to conventional culture on day 2, respectively (*p* < 0.001). The mRNA levels of *DDIT3* and *CA9* decreased by approximately 10 folds in 3D culture compared to conventional culture on day 2 (*p* < 0.001).

## 3. Discussion

The phenotype of cancer cells dedicating features such as chemoresistance, the rate of growth, morphology and mobility, is known to be greatly influenced by the microenvironment in which the cells exist. These findings suggest that it may be more biologically relevant to employ 3D culture models to study cancer biology [35]. In support of this concept, many studies comparing malignant epithelial or neurogenic cells cultured in 3D and those cultured conventionally have revealed substantial phenotypic and biochemical differences [36]. For instance, glioblastoma cells cultured in a 3D environment were found to have high levels of proliferation, invasiveness and IL-8 secretion when compared to the same cells grown in monolayer [4]. Furthermore, several studies have shown that experimental manipulations of cancer cells can generate vastly different results, depending on whether cells were cultured in 3D or conventionally. For example, inhibition of β-integrin was found to normalize the architecture of breast cancer cells, but only when the cells were cultured in 3D but not in monolayer [37]. There is direct evidence that results generated from using the 3D study models are more representative of the in vivo scenarios. As mentioned above, glioblastoma cell lines established from patient derived xenografts were found to have a drug resistant profile that correlated with the clinical outcome, but only if the cells were grown in a 3D environment [5]. Overall, it appears that studying cancer biology using 3D models is biologically and clinically relevant. 

To our knowledge, most of the published cancer studies using 3D models have focused on epithelial malignant cells (such as breast cancer) or neurogenic tumors (such as glioblastoma). In the field of MM research, we notice a relatively small number of studies on 3D culture. 3D dynamic devices such as bioreactor and microfluidic flow provide continuous nourishment to MM cells, but the equipment is expensive and does not allow high throughput drug screening [14,38]. In another model, de la Puente et al. employed cross-linked fibrinogen matrix supplemented by patient-derived bone marrow mononuclear cells and supernatants, and they successfully expanded fresh primary MM cells derived from 3 different patients [15]. However, the observation that there was a 2.5-fold increase in primary MM cells within 7 days appears to be inconsistent with the fact that the proliferation index of MM is generally low (i.e., average 6.5% Ki-67 positivity in Stage III MM patients) [39]. In our 3D culture system, which was adapted from Kirshner et al. [16,17], we employed a mixture of extracellular matrix proteins and Matrigel^®®^ designed to mimic the bone marrow matrix. This ‘reconstructed’ bone marrow matrix was previously shown to support the proliferation of primary MM cells for up to 30 days, a task that is difficult to achieve in conventional culture [16]. The value of this 3D system was further demonstrated in the same study that a relatively high dose of bortezomib eliminated only a subset of MM cells growing in the 3D culture [16], and this finding contrasted with the observation in conventional culture in previous studies in which nano-molar doses of bortezomib were sufficient to induce substantial cytotoxicity [40,41]. Importantly, as reported by Kirshner et al., the partial resistance to bortezomib seen in the 3D model was found to correlate with a poor clinical response to bortezomib monotherapy in MM patients [42,43]. In light of these findings, two more recent studies have adapted the same 3D model for assessment of the efficacy of novel anti-MM agents. Specifically, one study reported that anti-CD56-conjugated maytansinoid is able to overcome drug resistance in a co-culture system including MM cells and stromal cells [44]. In another study, two NF-κB inhibitors were found to induce cytotoxicity to putative MM cancer stem cells in the 3D model [45].

The biochemical effects of 3D culture on MM cells are not well understood. In this study, we have confirmed that the reconstructed bone marrow matrix can exert phenotypic and biochemical changes. Specifically, cells grown in this 3D culture system grew in large clusters instead of single cells or small clusters, as seen in the conventional culture system. In addition, the constitutive activation of STAT3 was observed in 3D cells but not in cells cultured conventionally. The upregulation of STAT3 was found to be rapid (i.e., within 24 h), and we have evidence that this biochemical abnormality is dependent on the continuous 3D culture, as STAT3 became inactive when MM cells were brought back to conventional culture. The mechanism of STAT3 activation in 3D is likely multi-factorial. First, as shown in this study, there were increases in the expression of cytokines (i.e., IL6, IL21 and IL10) known to activate STAT3 in MM. Second, the physical support of MM cells in 3D culture likely promotes whole-surface contact with extracellular matrix proteins, and this phenomenon leads to the 3D-induced spheriod formation and contributes to STAT3 activation. Similar observations were made in 3D cultured breast cancer cells [46]. We have found that the pSTAT3 nuclear staining was present in the vast majority of cells in the 3D culture, confirming that the elevated STAT3 activity is generalized phenomenon and not restricted to a small cell population. The 3D-induced upregulation of STAT3 activity may have contributed to the fact that the cell growth in the 3D environment caught up with that of conventional culture after a few days (i.e., Figure 1). In keeping with this concept, pharmacologic inhibition of STAT3 in MM-3D cells indeed resulted in a significant reduction in cell growth. The validity of these findings is also supported by the observation that MM cells in conventional culture were not sensitive to Stattic.

The oncogenic characteristics of STAT3 have been extensively reviewed [47,48,49,50]. In MM, STAT3 is believed to upregulate various proteins which are responsible for enhancing cell survival, proliferation and drug resistance [51,52,53]. STAT3 has been reported to be constitutively active in MM patients [22,54,55]. Pharmacological agents such as curcumin, piperlongumine, icaritin and LLL12 which blocked STAT3 phosphorylation were reported to suppress primary MM cell viability and/or MM tumor growth in animal models [22,56,57,58]. Clinically, a high pSTAT3 level has been reported to correlate with poorer progress-free survival and overall survival in newly diagnosed MM patients [59]. While constitutively high STAT3 activity was observed in >50% of primary MM samples, MM cell lines typically showed no evidence or a low level of STAT3 activity [22,60]. This discrepancy may result from the fact that various STAT3-activating cytokines and/or factors are abundant in vivo and in 3D culture models, but they are either absent or present in a low concentration in cell suspension. In this regard, extracellular matrix proteins, which are present in vivo and in 3D but not in cell suspension, have been found to be an important source of STAT3 activation in MM [61,62]. In support of this concept, MM cells cultured on fibronectin-coated surface had more robust IL6-induced STAT3 activation than those cultured in cell suspension [61]. In another study, it was found that MM cell lines showed STAT3 activation that could be enhanced by Reelin, an extracellular matrix protein [62]. Overall, there is ample evidence that extracellular matrix proteins contribute to the aberrant STAT3 activity in MM, and this phenomenon is recapitulated in 3D culture systems but not in cell suspension.

Our oligonucleotide array studies have revealed dramatic differences in the gene expression between MM-3D cells and cells cultured conventionally. Interestingly, *LPL* and *AGPNT2* (being significantly higher in MM-3D cells) as well as *DDIT3* (being significantly lower in MM-3D cells) are reported to be associated with STAT3 signaling. LPL, known to hydrolyze triglycerides into free fatty acids and glycerol, has been shown to be upregulated by STAT3 in chronic lymphatic leukemia (CLL) [63]. LPL is known to have oncogenic potential. As the result of the activity of LPL, it is believed that the generated free fatty acids binds to PPARα so as to promote the cell survival and proliferation of CLL cells [64]. ANGPT2 concentrations in bone marrow have been found to be significantly higher in patients with active MM compared to those with smoldering MM, MGUS or healthy donors [65]. ANGPT2 serum level is positively correlated with bone marrow microvascular vessel density in patients with active MM [65]. Additionally, it was found that bone marrow mononuclear cells from MM patients secreted substantially more ANGPT2 compared to those from healthy donors in a 3D bioreactor model [14]. DDIT3 was found to be decreased in MM-3D cells and has been reported to have tumor suppressor effects. DDIT3 is a protein that induces apoptosis in various types of cancer cells upon endoplasmic reticulum stress [66]. It is also reported that DDIT3 expression is suppressed by STAT3, leading to enhanced survival in mesothelioma [67]. 

## 4. Materials and Methods

### 4.1. Cell Lines, Patient Samples and Materials

Two human MM cell lines, U266 and RPMI8226 cells were obtained from Dr. Linda Pilarski. Karpas 299 and SupM2 cells were purchased from ATCC. All cell lines were grown in RPMI1640 medium supplied with 10% FBS with 1% streptomycin and penicillin except U266 cells, which were grown in RPMI1640 medium supplied with 15% FBS. Ficoll-Paque isolated bone marrow mononuclear cells from two MM patients and reconstituted bortezomib in sterile water (1 mg/mL) were obtained from Cross Cancer Institute, University of Alberta. Both patients #1 and #2 contained 10–20% monoclonal plasma cells according to their biopsy section. Stattic (Sigma, Oakville, ON, Canada) powder was dissolved in DMSO into 1 mg/mL solution. All procedures of patient sample handling were approved by Human Research Ethics Board, University of Alberta (#Pro00058140). Animal procedures for this study were approved by Animal Care and Use Committee, University of Alberta (#Pro00000282).

### 4.2. 3D Culture

The method for 3D culture was adapted from a previous publication [17]. In brief, 48-well plates were pre-coated with 100 μL of reconstructed endosteum (77 μg/mL fibronectin and 29 μg/mL collagen I in PBS) before seeding of 3D cultures. U266 or RPMI8226 cell pellets were resuspended first with 20 μL PBS. Matrigel^®^ (Corning, Corning, NY, USA), 1 mg/mL fibronectin and 2 mg/mL collagen IV were added to the resuspended cells in 4:2.5:1 ratio. 100 μL of cell matrix was loaded to each well and incubated at 37 °C for 1 h to allow polymerization. Finally, 1 mL of pre-warmed growth medium was added to the 3D culture. For recovery of 3D cells, 1 mL of cell recovery solution containing 5 mM EDTA, 1 mM sodium vanadate and 1.5 mM sodium fluoride was used. 

### 4.3. Preparation of Cells for Immunocytochemistry

The procedure of preparing U266-3D cells for immunocytochemistry is outlined in Figure S8 Histogel wells for each sample were created by inserting an Eppendorf tube into a well (24- or 48-well plate) with 400 µL of liquid histogel (Thermo Scientific, Ottawa, ON, Canada). Upon solidification of histogel, the Eppendorf tube was gently removed, leaving a concaved up and U-shaped well for 3D culture loading. The 3D cell culture was loaded into the well and allowed to solidify for 1 h at 37 °C. ~300 µL of growth medium was added to the 3D cell culture and incubated for two days. On the day of embedding, the growth medium was removed and 200 µL of liquid histogel was added on top to encapsulate the 3D cell culture within the histogel. The entire histogel was then fixed in 4% formaldehyde at 4 °C overnight and processed for paraffin wax embedding. For U266 and Karpas 299 cells, 2 × 10^6^ cells were pelleted, resuspended in 100 µL histogel, transferred to a 7 × 7 × 5 mm plastic mold (Simport) and fixed in 4% paraformaldehyde for embedding. For U266 xenograft cells, 5 × 10^5^ U266 cells stably transduced with luciferase gene were injected into a severe combined immunodeficient diabetic (SCID) mouse intravenously via tail vein. The mouse was euthanized when it became immobile and lost more than 20% body weight. The total bone marrow cells were isolated from the femur, resuspended in 100 µL histogel and transferred to a plastic mold for embedding. All of isolated bone marrow cells were confirmed to be U266 cells by bioluminescence imaging. After embedding, processing and sectioning, the sample slides were rehydrated in xylene and decreasing concentrations of ethanol. The antigens were retrieved using 1× citrate buffer (Sigma) by microwaving in a pressure cooker for 20 min. The pSTAT3 antibody (Santa Cruz, clone B-7, Mississauga, ON, Canada) was diluted as 1:50 in antibody diluent (DAKO). MACH2 mouse HRP polymer (Biocare Medical) was used as a secondary antibody. The chromogen and substrate were mixed and applied to each slide for 2 min for color development (DAKO).

### 4.4. DNA Pulldown Assay

Cell pellets from MM or MM-3D cells were lysed with CellLytic M (Sigma) with protease inhibitor and phosphatase inhibitor cocktails (Millipore, Etobicoke, ON, Canada) on ice for 30 min. 300 µg of total cell lysate was mixed with 3 pmol of STAT3 DNA probe (Biotin-5′-GATCTAGGAATTCCCAGAAGG-3′) for 30 min on a rotator at room temperature. 75 µL of streptavadin agarose beads (Thermo Scientific, Ottawa, ON, Canada) was added to the DNA-lysate mix. The whole solution was incubated on a rotator at 4 °C overnight. The beads were washed three times with ice-cold PBS. SDS loading buffer was added to beads and boiled for 5 min to dissociate bound proteins. The beads were spun down and the supernatant was subject to SDS-PAGE. 

### 4.5. Cellular Thermal Shift Assay (CETSA)

The original protocol of CETSA was followed [34]. In brief, both MM and MM-3D cells were cultured for 48 h. Cells were harvested using cell recovery solution and incubated on ice for 1 h with brief vortex every 15 min. Cells were pelleted and washed once with cold and sterile PBS. Cells were resuspended in PBS supplied with 5% protease inhibitor cocktail and 2.5% PMSF prior to heating. Resuspended cells were heated at 54 °C for U266 cells and 52 °C for RPMI8226 cells for 3 min using a thermal cycler. Cells were lysed by three freeze-thaw cycles in liquid nitrogen. Aggregated proteins were precipitated at 20,000 *g* for 20 min at 4 °C. The supernatant was collected, heated (70 °C for 10 min) and dissolved in 4× SDS loading buffer prior to SDS-PAGE.

### 4.6. Cell Viability and Apoptosis Assays

Both MM and MM-3D cells on 48-well plates after drug treatment were recovered by cell recovery solution and resuspended in fresh growth medium. 100 µL resuspended cells were transferred to a 96-well plate. Cell viability was measured by CellTiter 96^®^ AQueous One Solution Cell Proliferation Assay (i.e., MTS assay, Promega, Madison, WI, USA) or trypan blue exclusion assay (Amresco, Solon, OH, USA). The apoptosis was measured by following the instructions of FITC Annexin V Apoptosis Detection Kit I (BD Biosciences, San Jose, CA, USA).

### 4.7. Oligonucleotide Array

Total RNA of both U266 and U266-3D cells were prepared using RNeasy Mini Kit (Qiagen, Germantown, MD, USA). First strand cDNA was synthesized using RT2 First Strand Kit (Qiagen). All PCR reactions were prepared by adding cDNA, RT2 SYBR Green ROX qPCR Mastermix (Qiagen) into the 96-well plates of RT2 Profiler Human Cancer PathwayFinder PCR Array (Qiagen). The array contains 84 representative genes which are responsible for 9 biological pathways which are complicated in human cancers. The cycle threshold (CT) values were obtained and standardized using the CT value of GAPDH. The logarithmic ratio of mRNA expression fold changes (3D to 2D) for each gene was calculated and ranked from highest to lowest.

### 4.8. Reverse Transcriptase Polymerase Chain Reaction (RT-PCR)

The total RNA of U266 cells in conventional culture for 2 days or in 3D culture for 1 to 4 days were extracted using RNeasy Plus Mini Kit (Qiagen). First strand cDNA was prepared using SuperScript^®^ Reverse Transcriptase kit (Invitrogen). RT-PCR reactions were prepared using SYBR^®^ Select Master Mix (Applied Biosystems, Foster City, CA, USA). The sequences of all forward and reverse primers used in this study are summarized in Table 1. The fluorescence signal was detected and measured by 7900HT Fast Real-Time PCR System and analyzed by SDS2.3. The gene expression was normalized to GAPDH.

### 4.9. Western Blot Analysis

Both MM and MM-3D cell pellets were lysed by 1× RIPA buffer (Millipore) with protease inhibitor and phosphatase inhibitor cocktails (Millipore) on ice for 30 min. Protein concentration of each lysate was measured using BCA protein assay kit (Thermo Scientific). Equal amount of protein was loaded on 10% homemade polyacrylamide gels for SDS-PAGE at 100 volts. Proteins in polyacrylamide gel were transferred to nitrocellulose membrane (Bio-Rad, Hercules, CA, USA) at 100 V for 2 h. Primary antibodies used were anti-pSTAT3 (Y705) (1:2000, CST, #9145), anti-STAT3 (1:1000, CST, #124H6), anti-pErk (T202/Y204) (1:2000, CST, #4377), anti-Erk (1:1000, Enzo, #ADI-KAP-MA001), anti-pAkt (S473) (1:1000, CST, #4060), anti-Akt (1:1000, CST, #9272), anti-cleaved (V1744) Notch1 (1:1000, CST, #4147), anti-Notch1 (1:1000, CST, #3439), anti-pIKBα (S32) (1:1000, CST, #9241), anti-IKBα (1:1000, CST, #4812), anti-β-actin (1:1000, CST, #58169), anti-PARP (1:1000, CST, #9532) and anti-Caspase3 (1:1000, CST, #9665). Secondary antibodies used were HRP-conjugated anti-mouse (1:2000, CST, #7076) and anti-rabbit (1:2000, CST, #7074). Signals on the membrane were developed using Pierce™ ECL Western Blotting Substrate (Thermo Scientific) and exposed to X-ray films (Fuji, Tokyo, Japan).

### 4.10. Statistical Analysis

All numerical data in this study was presented as the mean from experiment replicates or independent experiments as described in the figure legends. Statistical significance between groups were analyzed using Student’s *t*-test with α = 0.05 except Figure 6, for which one-way ANOVA with Dunnett’s multiple *t*-test (α = 0.05) were employed. The analysis was done using Microsoft Excel 365 except Figure 6, for which GraphPad Prism 7 was used for analysis.

## 5. Conclusions

Our studies have revealed that culturing MM cells in our 3D, reconstructed bone marrow model consistently and effectively induces STAT3 activation in MM cells, and this biochemical aberrancy mimics the majority of primary MM patient samples and in vivo xenografted MM cells. These observations suggest that the use of 3D culture systems to study MM is biologically and clinically meaningful. Our study results also suggest that further evaluation of the therapeutic efficacy of anti-STAT3 agents should be done in vivo or in 3D culture systems. The value of anti-STAT3 therapeutic agents in treating MM can be further highlighted by its additive or synergistic effectiveness with bortezomib. Lastly, since our protocol has greatly facilitated immunocytochemical studies of MM-3D cells, it may be useful to investigate MM-stromal cell interactions in the future.

## Figures and Tables

**Table 1:** Forward and reverse primers used in this study.

Gene	Forward Primer	Reverse Primer
*IL6*	5′-TCCAGTTGCCTTCTTGGGAC-3′	5′-GTACTCCAGAAGACCAGAGG-3′
*IL21*	5′-TGTGAATGACTTGGTCCCTGAA-3′	5′-AACAGGAAAAAGCTGACCAC-3′
*IL10*	5′-GCCTAACATGCTTCGAGATC-3′	5′-TGATGTCTGGGTCTTGGTTC-3′
*LPL*	5′-ACAAGAGAGAACCAGACTCCAA-3′	5′-GCGGACACTGGGTAATGCT-3′
*ANGPT2*	5′-AACTTTCGGAAGAGCATGGAC-3′	5′-CGAGTCATCGTATTCGAGCGG-3′
*DDIT3*	5′-GGAAACAGAGTGGTCATTCCC-3′	5′-CTGCTTGAGCCGTTCATTCTC-3′
*CA9*	5′-GGATCTACCTACTGTTGAGGCT-3′	5′-CATAGCGCCAATGACTCTGGT-3′
*GAPDH*	5′-GGTCTCCTCTGACTTCAACAGCG-3′	5′-ACCACCCTGTTGCTGTAGCCAA-3′

## Supplementary Materials

The following are available online at http://www.mdpi.com/2072-6694/10/6/206/s1, Figure S1. MM-3D cells form larger cell spheroids. Figure S2. MM cells cultured conventionally do not acquire STAT3 activity. Figure S3. STAT3-activating cytokines are upregulated in U266-3D cells. Figure S4. Increased seeding number results in less sustained pSTAT3 level in 3D culture. Figure S5. Stattic does not induce apoptosis in conventionally cultured MM cells. Figure S6. Stattic does not improved bortezomib-induced cytotoxicity in MM cells cultured conventionally. Figure S7. Gene expression changes in MM-3D cells are STAT3-relevant. Figure S8. Schematic procedure of immunocytochemistry of MM-3D cells.
