# Supplementary material for: Constitutive Activation of STAT3 in Myeloma Cells Cultured in a Three-Dimensional, Reconstructed Bone Marrow Model
Source: Cancers (Basel). 2018 Jun 16;10(6):206. doi: 10.3390/cancers10060206 (PMC6024941; doi:10.3390/cancers10060206)
Supplement: Supplementary file 1 [file cancers-10-00206-s001.docx]

**Supplemental Document**

**
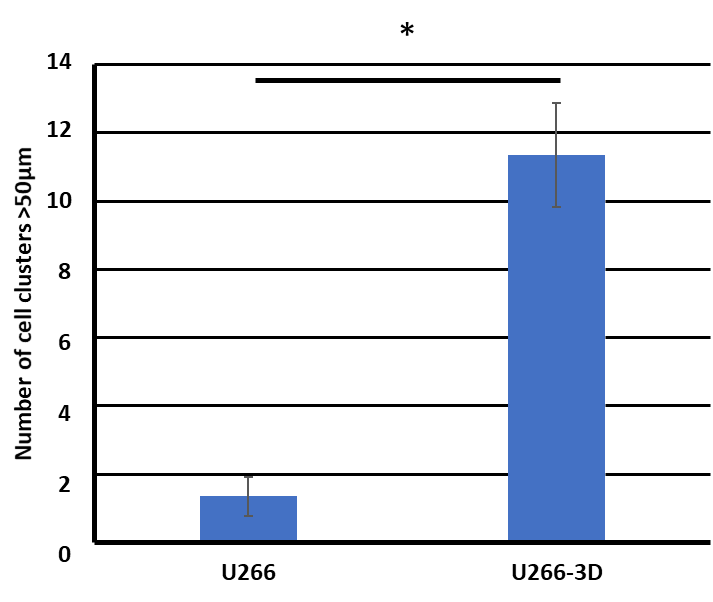
**

**Figure S1. MM-3D cells form larger cell spheroids.** The number of cell clusters with the greatest dimension >50 µm from three different microscopic images of U266 and U266-3D cells were counted. The images were of 100X magnification. The error bars represent the standard deviation from three different fields, *p<0.05, Student’s t-test.


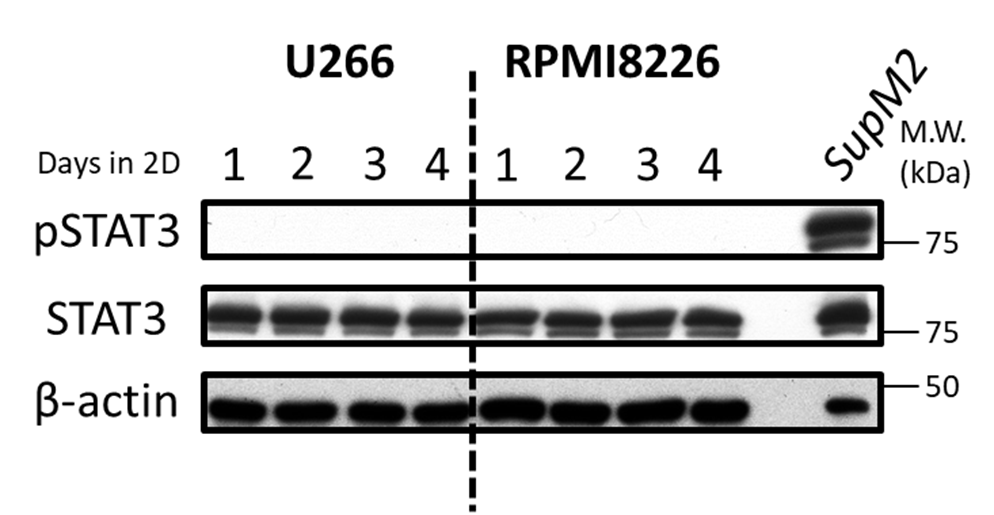


**Figure S2. MM cells cultured conventionally do not acquire STAT3 activity.** The STAT3 activity of U266 and RPMI8226 cells in conventional (2D) culture from day 1 to day 4 were examined by Western blot analysis of pSTAT3 levels. β-actin was probed as a loading control. SupM2 cell lysate was also included as a positive control for pSTAT3 level. 2.5x10^5^ cells were seeded initially.**
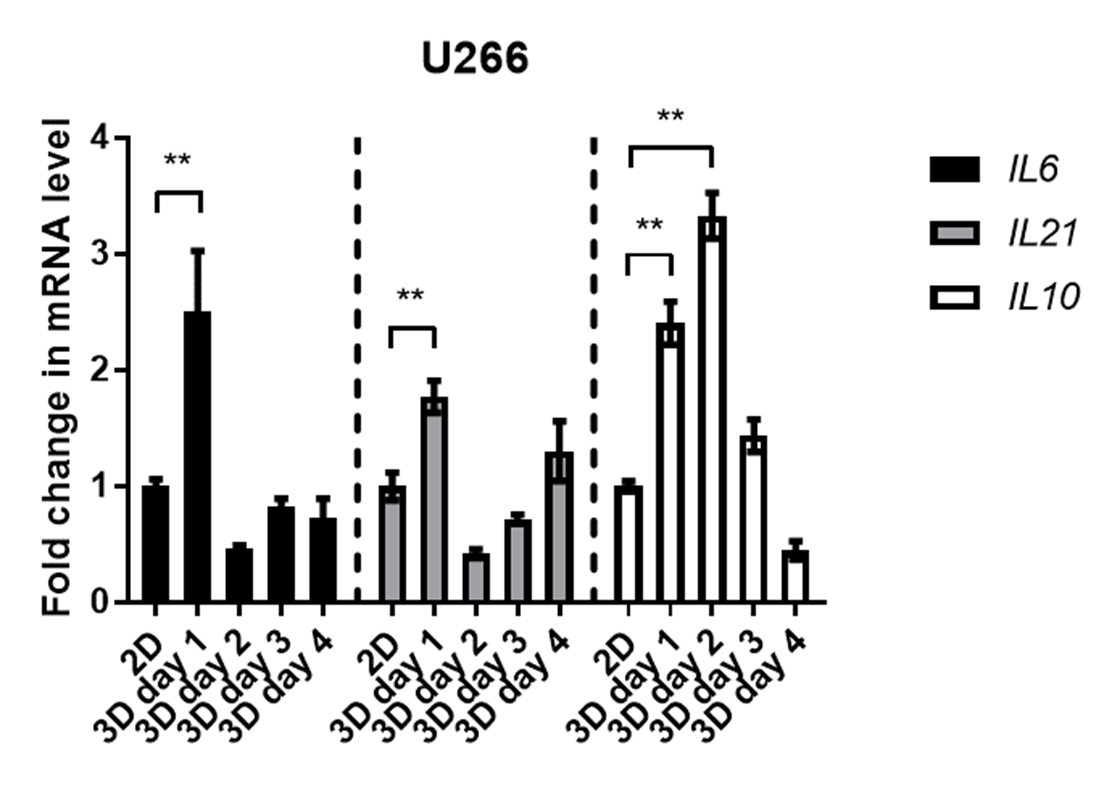
**

**Figure S3. STAT3-activating cytokines are upregulated in U266-3D cells.** Quantitative RT-PCR of *IL6* (black), *IL21* (grey) and *IL10* (white) mRNA levels in U266 cells in conventional culture (2D) or day 1 to 4 in 3D culture. 2.5x10^5^ cells were seeded initially. The primers used for each gene are described in Materials and Methods. The error bars represent standard deviation from a triplicate experiment. **p<0.001 compared to 2D, one-way ANOVA with Dunnett’s multiple t-test.

**
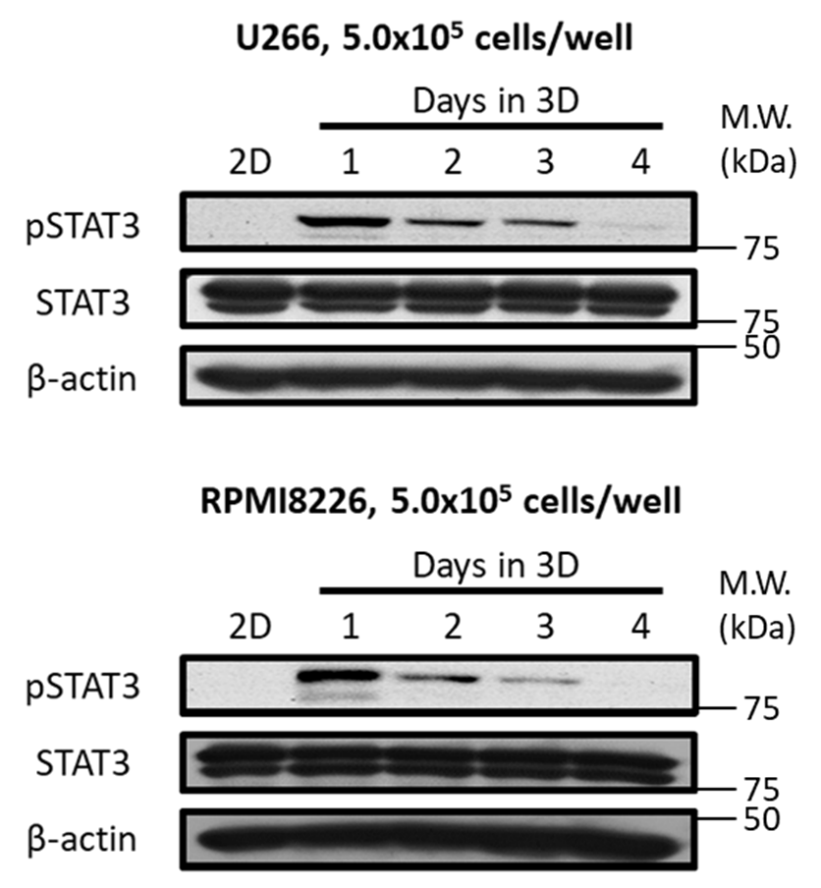
**

**Figure S4. Increased seeding number results in less sustained pSTAT3 level in 3D culture.** Western blot analysis of pSTAT3 and STAT3 levels of U266 and RPMI8226 cells in 2D or 3D culture from day 1 to day 4 with a higher cell concentration (5.0x10^5^ cells). β-actin was probed as a loading control.

**
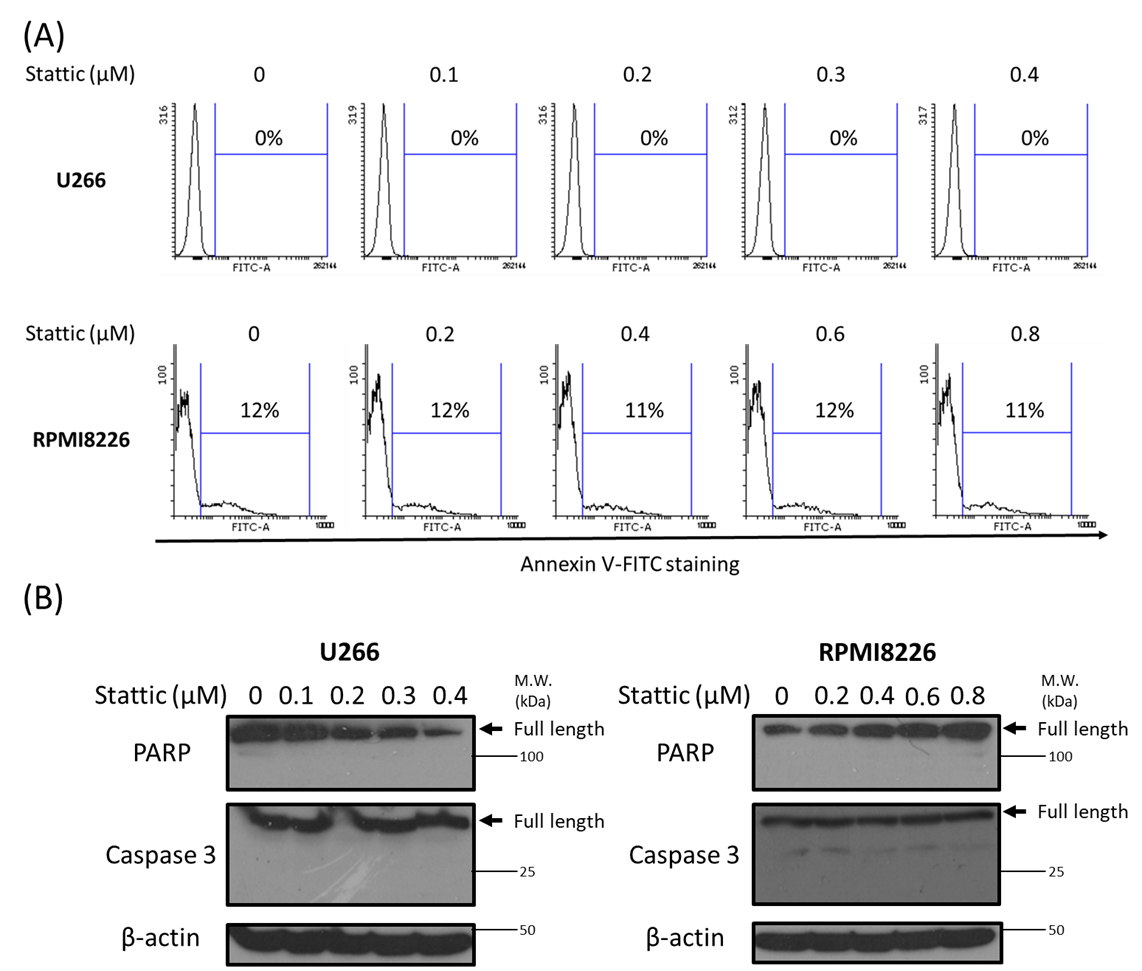
**

**Figure S5. Stattic does not induce apoptosis in conventionally cultured MM cells.** (A) The effect of STAT3 inhibition on apoptosis in U266 and RPMI8226 cells in conventional culture. The cells were treated with Stattic for 24 hours and stained with an apoptotic marker Annexin V. The percentage of Annexin V-positive cells was analyzed by flow cytometry. (B) The expression levels of two apoptotic markers, cleaved PARP and cleaved caspase 3, in U266 and RPMI8226 cells cultured conventionally after 24 hours of Stattic treatment were examined by Western blot analysis. β-actin was probed as a loading control. For all the experiments above, U266 and RPMI8226 cells were cultured for 2 and 1 days before the Stattic treatment to reach a substantial pSTAT3 level, respectively. 2.5x10^5^ cells were seeded initially.

**
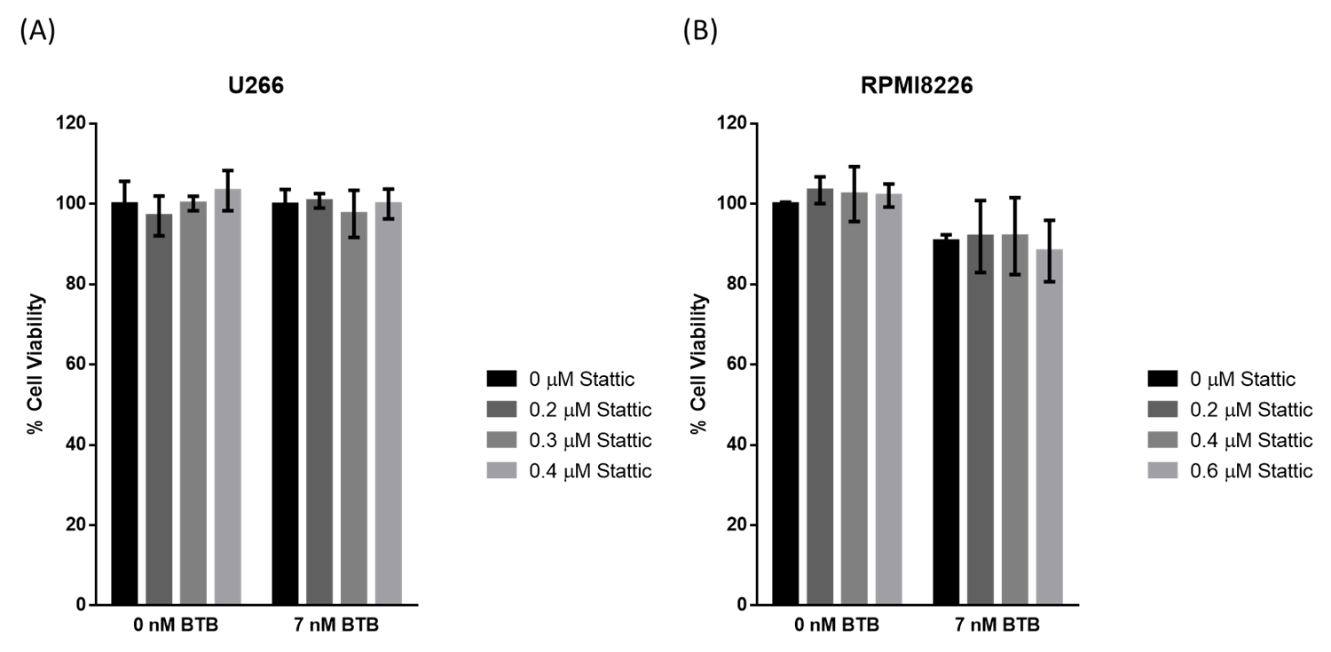
**

**Figure S6. Stattic does not improved bortezomib-induced cytotoxicity in MM cells cultured conventionally.** Cell viability of U266 and RPMI8226 cells in conventional culture was measured after treatment with Stattic, bortezomib (BTB) or both for 48 hours. U266 and RPMI8226 were pre-cultured for 2 days and 1 day before drug treatment, respectively. Cell viability was measured by MTS assay and normalized to the cell viability of untreated cells. 2.5x10^5^ cells were seeded initially. The error bars represent standard deviation from a triplicate experiment, **p<0.001, Student’s t-test.

**
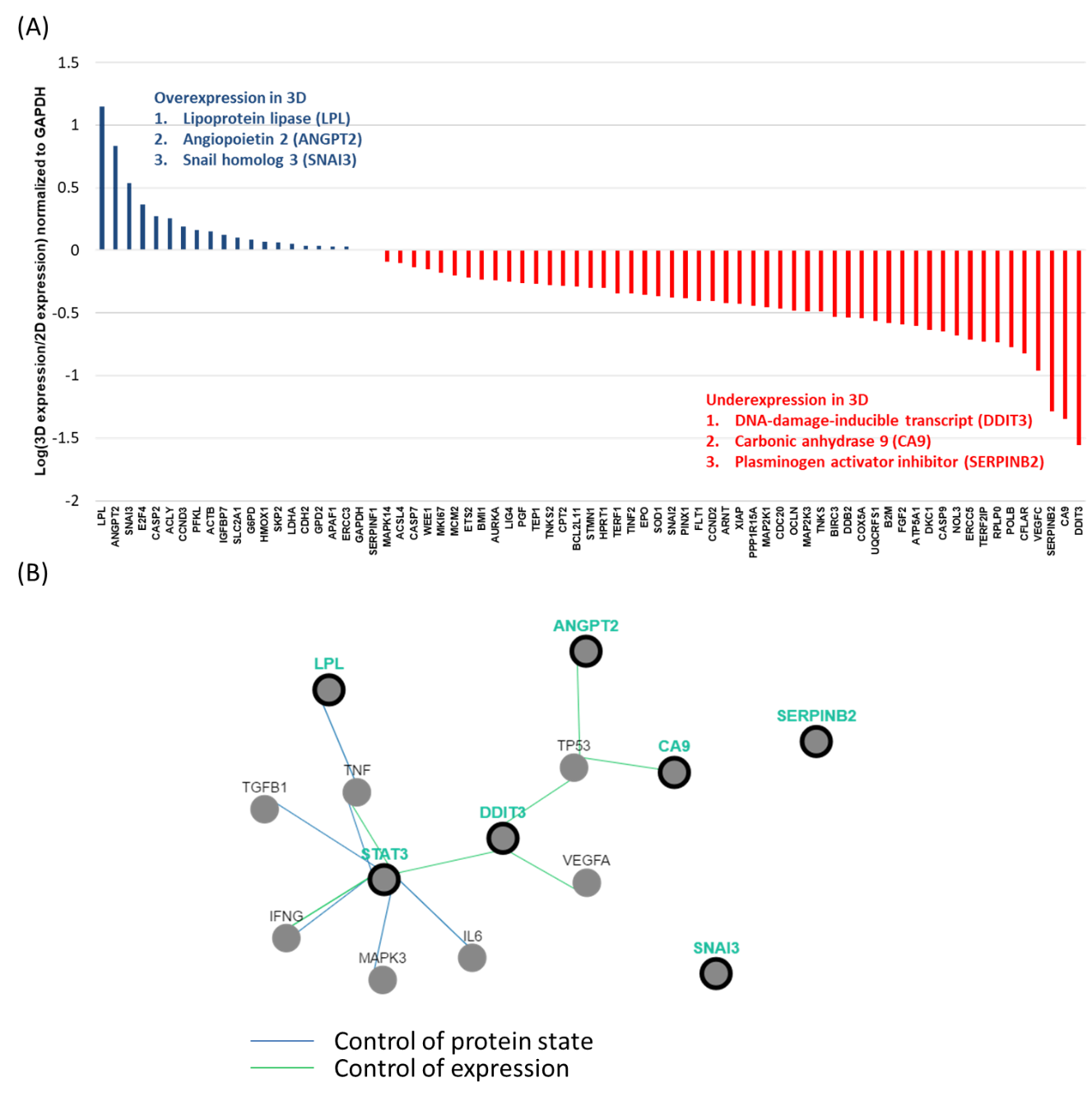
**

**Figure S7. Gene expression changes in MM-3D cells are STAT3-relevant.**  (A) Oligonucleotide assay of human cancer-related genes using U266 or U266-3D cDNA. The cDNA was extracted from U266 cells after 2 days in conventional (2D) or 3D culture. All 2D and 3D gene expressions were normalized to the corresponding *GAPDH* expressions. The logarithm of gene expression ratio (3D/2D) was computed for each gene and ranked from the highest (left) to the lowest (right). The top three most upregulated (blue) and downregulated (red) genes were indicated in the graph. (B) Signaling pathway analysis of the top three most upregulated and downregulated genes from (A) and their relationship with STAT3 using Pathway Common Network Visualizer. A blue line connects two genes which one can affect the expression level of the other. A green line connects two genes which one can affect the protein state of the other.

**
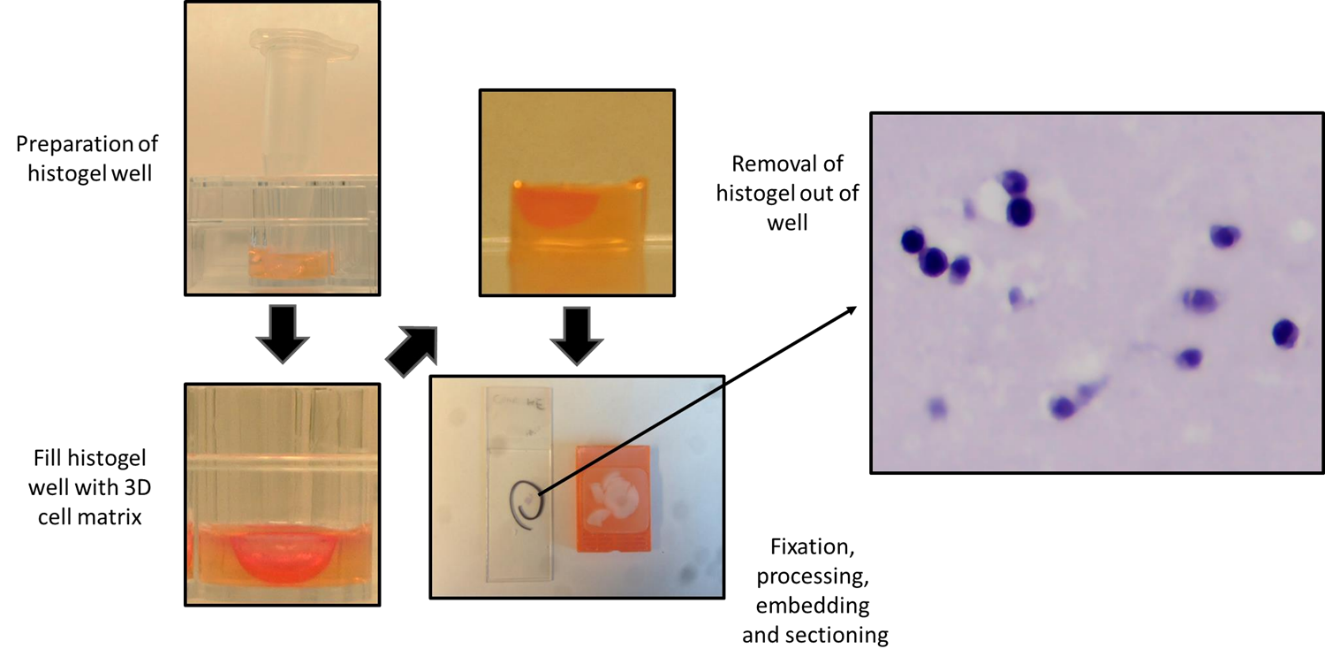
Figure S8. Schematic procedure of immunocytochemistry of MM-3D cells.** A 1.5 ml Eppendorf tube was inserted into liquid histogel in a 48-well plate. Upon solidified, the “histogel well” was loaded with the 3D cell matrix containing Matrigel^®^, fibronectin, collagen IV and MM cells. After solidification of 3D cell matrix, growth medium was applied on top of 3D cell matrix. On the day of harvest, the entire histogel well was scooped out of plate with a surgical knife and transferred to a tissue cassette for fixation, embedding, processing and sectioning. An image of H&E staining of MM-3D cells following this protocol was depicted on right side.
